# Open field study on the efficacy of oral fluralaner for long-term control of flea allergy dermatitis in client-owned dogs in Ile-de-France region

**DOI:** 10.1186/s13071-016-1463-z

**Published:** 2016-03-23

**Authors:** Odile Crosaz, Elodie Chapelle, Noëlle Cochet-Faivre, Diane Ka, Céline Hubinois, Jacques Guillot

**Affiliations:** Department of Parasitology, Mycology and Dermatology, CHUVA, École nationale vétérinaire d’Alfort, UPE, Maisons-Alfort, 94704 France; MSD Animal Health, Beaucouzé, France

**Keywords:** Fluralaner, Flea, *Ctenocephalides felis*, Flea allergy dermatitis, Long-term control

## Abstract

**Background:**

Fluralaner is the first orally administered isoxazoline to provide 12 weeks of activity against fleas and ticks after a single administration. As a result of its potent anti-flea activity, oral fluralaner may be proposed as a component of a strategy for the control of flea allergy dermatitis (FAD) in dogs. The open field study reported here assessed the efficacy of fluralaner for long-term control (up to 6 months) of FAD in affected client-owned dogs maintained under common household conditions in the Ile-de-France region.

**Methods:**

This was an open pre-treatment versus post-treatment study. Client-owned dogs with clinical signs of FAD were recruited and treated with oral fluralaner (Bravecto®) at 25-56 mg/kg body weight on days 0 and 84. The dogs’ condition was assessed at each visit (on days 0, 28, 84 and 168) using the following three parameters: (i) extent of skin lesions based on the scoring system for canine FAD; (ii) pruritus severity based on the pruritus visual analog scale; (iii) presence or absence of fleas or flea feces.

**Results:**

Of the 26 dogs initially enrolled, 23 were presented on day 28, 20 on day 84 and 16 for the final evaluation on day 168. Eighteen out of 20 dogs (90 %) presented on day 84 and 15 out of 16 dogs (94 %) presented on day 168 showed a complete clinical resolution. The post-treatment FAD clinical scores on days 28, 84 and 168 were significantly different from that of the pre-treatment with a reduction of 89.8 %, 98.8 % and 99.8 %, respectively. The post-treatment pruritus index values on days 28, 84 and 168 were significantly different from that of the pre-treatment with a reduction of 45.2 %, 71.2 % and 80.8 %, respectively.

**Conclusions:**

The present study confirmed that oral fluralaner treatment should be considered as effective for long-term control of clinical signs in FAD affected dogs.

**Electronic supplementary material:**

The online version of this article (doi:10.1186/s13071-016-1463-z) contains supplementary material, which is available to authorized users.

## Background

*Ctenocephalides felis* is the main flea species infesting both dogs and cats. It also represents the most important ectoparasite for dogs in many parts of the world. Without treatment, a persistent flea infestation can induce intense pruritus and self-inflicted trauma. With repeated exposure, dogs may develop hypersensitivity to components of flea saliva, a “condition” that leads to flea allergy dermatitis (FAD) [1, 2]. Dogs that are predisposed to FAD and live in a flea endemic area will typically develop clinical signs by the age of 5 years. However, clinical signs can develop at any age, particularly if animals move from a low-exposure risk area to an area where fleas are endemic. Once sensitization has occurred, clinical relapse can be initiated by a small number of flea bites [3].

In dogs, the typical clinical presentation of FAD is a pruritic papular dermatitis that is concentrated on the rump, dorsal thorax, flanks, tail, and perineal area. Pruritus is usually focused on the caudal areas and tail, although some dogs express generalized pruritus. Clinical signs can include alopecia, crusts, hyperpigmentation, lichenification and pyotraumatic dermatitis lesions. Secondary skin infections frequently complicate the clinical picture of dogs, which can develop bacterial folliculitis and/or *Malassezia* dermatitis [1–3].

The isoxazolines are a novel class of antiparasitic drugs that inhibit gamma-aminobutyric acid (GABA) and glutamate-gated chloride channels with significant selectivity for insect neuron receptors compared with mammalian receptors. This results in excess neuronal stimulation and arthropod death. To date, this new class includes three molecules: afoxolaner, fluralaner, sarolaner, which are all formulated for prescription by veterinarians as flavored chewable tablets for dogs. Fluralaner is the first orally administered active ingredient (Bravecto®, MSD Animal Health, Madison NJ) to provide 12 weeks of insecticidal activity against fleas and up to 12 weeks of acaricidal activity against important tick genera after a single administration. Following oral administration to dogs, fluralaner is rapidly absorbed and provides 100 % effectiveness against fleas and ticks within 12 h. Blood levels are then sustained and provide a high level of flea and tick killing activity for up to 12 weeks [4, 5]. Oral fluralaner has shown efficacy as a component of a strategy for control of FAD [6–8]. A polymer matrix collar containing a combination of 10 % imidacloprid and 4.5 % flumethrin has been licensed for use in dogs and cats and this collar confers a long-term (8 months) protection against fleas (and ticks).

The open field study reported here assessed the efficacy of fluralaner for long-term control (up to 6 months) of FAD in affected client-owned dogs maintained under common household conditions in the Ile-de-France region.

## Methods

This was an open pre-treatment versus post-treatment clinical field study. The study was conducted in the Small Animal Hospital of Alfort Veterinary College (CHUVA, France) from June 2014 to July 2015. Cases were recruited from the canine patient population presented for examination at the dermatology unit of the hospital.

Client-owned dogs diagnosed with FAD were enrolled in the study with written informed consent from their owners. All dogs came from Ile-de-France region and were each enrolled independently. During the study, dogs were kept at home by their owners and fed and exercised according to their usual routine.

Dogs were diagnosed with FAD on the basis of clinical signs consistent with published descriptions [9, 10] and exhibited both pruritus and typical FAD lesions were noticed. Lesions compatible with FAD included: erythema, papules, alopecia, crusts, lichenification and hyperpigmentation in specific areas (dorsolumbar, ano-genital, tail), and/or pyotraumatic dermatitis.

Dogs were excluded from the study if: (i) they had been treated with an external antiparasitic treatment within the 30 days before presentation; (ii) they were suspected to have sarcoptic mange or showed a positive otopodal reflex; (iii) they were suspected to have canine atopic dermatitis (CAD). CAD diagnosis was based on the following clinical criteria [9]: facial lesions, otitis, pododermatitis or carpal or tarsal dermatitis. The absence of flea or flea feces was not included in exclusion criteria. Dogs were not included if it was not possible to treat all animals in the household against fleas.

During the first visit (day 0), the clinical history was collected for each dog and a standard clinical examination was performed. The owner was requested to present each enrolled dog for clinical evaluation on days 0, 28 (D0 + 1 month), 84 (D0 + 3 months) and 168 (D0 + 6 months). The dog’s condition was assessed at each visit using the following three parameters:

1. Skin lesions were measured by a veterinary dermatologist using the scoring system for canine FAD (SSCFAD, based on Laffort-Dassot et al. [9]). This scoring system is based on evaluation of 6 primary and secondary lesions (erythema, papules, excoriations, alopecia, kerato-seborrheic dermatitis and lichenification) at each of 6 body areas (dorsolumbar area, lateral area, hind limb, ano-genital area, front-ventral area and hind ventral area). Each lesion is graded from 0 (no clinical sign) to 10 (severe signs). The minimum possible score is 0, and the maximum possible score (indicating the most severe skin lesions) is 360.
2. Pruritus severity was assessed by the dog owner using a validated pruritus visual analog scale (PVAS) [11] using a score from 0 to 10.
3. Presence or absence of fleas or flea feces. Fleas were counted if they were observed.

Each dog was treated with a Bravecto® tablet administered according to the SPC recommended dose based on the dog’s weight on day 0 and again on day 84 (D0 + 3 months). Included dogs received no concomitant treatment with any other flea-control drugs or with any drug with antipruritic or anti-inflammatory activity throughout the 6-month study. Antiseptic or antibiotic treatments were allowed only during the first month of the study. Cohabiting animals residing at study households also received an antiparasitic treatment either with indoxacarb spot-on (Activyl®) for cats (every month), or oral fluralaner (Bravecto®) for dogs (every 12 weeks). Owners were asked to report any observed health issues or adverse events following treatment. An antiparasitic spray/fogger could be used for the house, at the discretion of the veterinarian.

The SSCFAD reduction was calculated at each time point *t* using the SSCFAD arithmetic mean in the following formula:

$$ \mathsf{SSCFAD}\ \mathsf{reduction}\ \left(\%\right) = \mathsf{100} \times \left(\mathsf{mean}\ \mathsf{day}\ \mathsf{0}\ \hbox{--}\ \mathsf{mean}\kern0.5em \mathit{\mathsf{t}}\right)\kern0.1em /\mathsf{mean}\ \mathsf{day}\ \mathsf{0}. $$

The PVAS reduction was calculated at each time point *t* using the arithmetic mean of pruritus scale according to the following formula:

$$ \mathsf{PVAS}\ \mathsf{reduction}\ \left(\%\right) = \mathsf{100} \times \left(\mathsf{mean}\ \mathsf{day}\ \mathsf{0}\ \hbox{--}\ \mathsf{mean}\kern0.5em \mathit{\mathsf{t}}\right)\kern0.1em /\mathsf{mean}\ \mathsf{day}\ \mathsf{0}. $$

SSCFAD and PVAS values were analyzed by a mixed linear model including day as a fixed effect. Kenward-Roger correction was used to determine the denominator degrees of freedom [12]. Least squares means were used for treatment comparisons.

The null hypothesis was that there was no significant difference in the testing parameter between the pre-treatment and post-treatment. Two tailed tests were used for the comparison. Statistical significance was declared when P ≤ 0.05. The primary software was SAS version 9.3 (SAS Institute Inc., Cary, NC, USA).

## Results

Twenty-six client-owned dogs with suspected FAD were enrolled in the study. They were mixed and pure breed, ranging from 1 to 14 years old, and weighing between 4.5 and 40 kg. There were 11 females and 15 males. Four breeds were more frequently represented: Maltese (4), Jack Russell Terrier (4), Labrador Retriever (3) and Shih Tzu (3). Fourteen dogs lived in a house with access to a garden while 12 exclusively lived in an apartment. Fourteen of the enrolled dogs were the only animal of the house, while 12 lived with at least one other pet (Additional file 1).

Five of the 26 enrolled dogs received a topical antiseptic treatment during the first month, and nine received antibiotics (sometimes combined with topical antiseptic treatments). A spray formulation (containing permethrin and (S)-methopren) (Tiquanis®) was used for the control of environmental stages of fleas in five cases. Of the 26 dogs enrolled on day 0, 23 were presented on day 28, 20 on day 84 and 16 for the final clinical evaluation, on day 168.

**Fig. 1:**
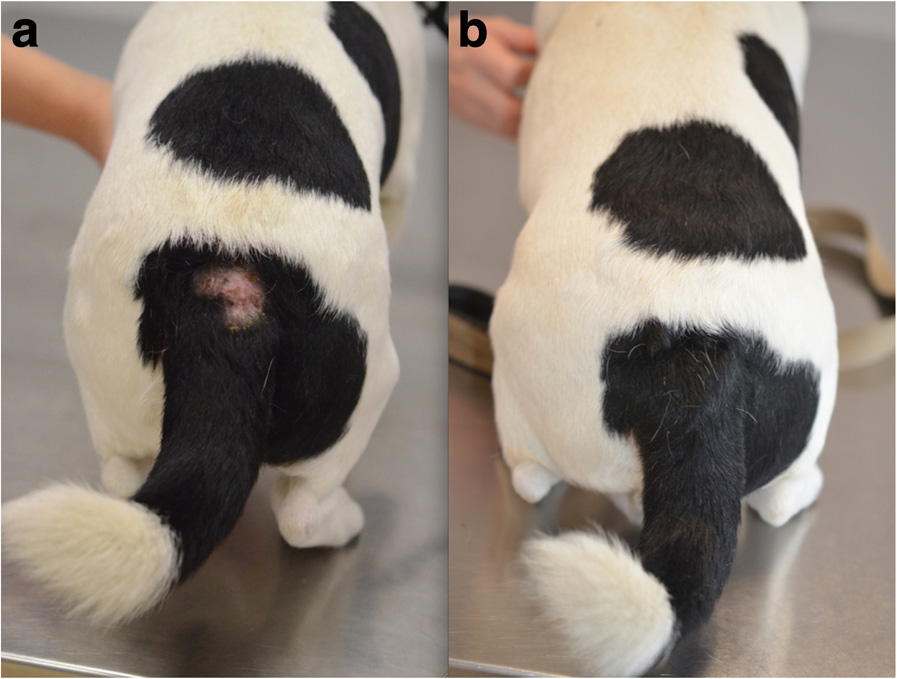
A 6 year-old Jack Russell Terrier presented with a dorsolumbar lesion at day 0 (**a**) and the same dog at day 84 (**b**) showing complete resolution of the lesion

Clinical examination on the first visit found that dogs mostly showed lesions in two body areas: the dorso-lumbar area and the ano-genital area. Eighteen out of 20 dogs (90 %) presented on day 84 and 15 out of 16 dogs (94 %) presented on day 168 showed complete clinical resolution. A significant improvement in clinical signs was observed for all the dogs over the study period (Table 1 and Fig. 1). The post-treatment FAD clinical scores on days 28, 84 and 168 were significantly different from that of the pre-treatment on day 0 (*P* values < 0.0001 for all 3 post-treatment days) with a reduction of 89.8 %, 98.8 % and 99.8 %, respectively. The FAD clinical scores on post-treatment days 28, 84 and 168 were not significantly different (*P* = 0.3651 for the difference between day 28 and day 84; *P* = 0.3511 for the difference between day 28 and day 168; *P* = 0.9363 for the difference between day 84 and day 168) from each other.

**Table 1:** Clinical scores assessed by a veterinary dermatologist and pruritus severity assessed by owners in FAD-affected dogs following oral fluralaner treatment

Parameter	D0	D28	D84	D168
Number of dogs	26	23	20	16
Mean SSCFAD-score values	54	5.5	0.6	0.1
Mean pruritus scale values	7.3	4	2.1	1.4
P value compared with D0		< 0.0001	< 0.0001	< 0.0001

The owner assessment of pruritus decreased significantly over the same period (Table 1). The post-treatment pruritus visual analogue scales on post-treatment days 28, 84 and 168 were significantly different from that of the pre-treatment (P values < 0.0001 for all 3 post-treatment days) (Additional file 2), with a reduction of 45.2 %, 71.2 %, and 80.8 %, respectively. The PVAS values on post-treatment days 84 and 168 were significantly different from that on the post-treatment day 28 (*P* = 0.0031 for the difference between day 28 and day 84; *P* = 0.0002 for the difference between day 28 and day 168), but were not significantly different from each other (*P* = 0.2963). The SSCFAD reduction and the pruritus score reduction were not affected by whether the dog had access to a garden or lived with another pet in the household (data not shown).

The flea count mean was 1.38 on day 0; this decreased to 0.00 on day 28 and remained at 0.00 on days 84 and 168.

An antiparasitic spray was used in the house (on day 0) in only five of the cases (17 %). Due to this small number, it was not possible to show any difference in response when comparing houses that were fogged and those that were not fogged.

No adverse clinical event was observed in any dog after either of the two oral fluralaner administrations at days 0 and 84.

## Discussion

This is the first report to show long-term successful management of FAD, as enrolled dogs were requested to return for follow-up examinations at a final visit 168 days (6 months) following the first treatment. This report was an open study because both investigators and owners knew which treatment was being administered to the dogs. An untreated control group was not included for ethical reasons. To date, there are only few clinical field studies published regarding treatment strategies for controlling FAD in dogs [7, 8, 13]. Rohdich et al. [7] conducted a randomized, multi-centered field European study to compare the flea- and tick-control efficacy for dogs over a 12-week period with either a single oral dose of fluralaner or with three sequential topical fipronil treatments. Of dogs showing clinical FAD at the study start, 85.7 % (30 out of 35) in the fluralaner-treated group and 55.6 % (10 out of 18) in the fipronil-treated group were evaluated at each time point as showing no clinical signs of FAD until study completion. Fisara et al. [13] evaluated the clinical response to topical indoxacarb treatment every 4 weeks for 12 weeks in 25 client-owned dogs with FAD in Australia [12]. Of the 24 dogs that completed the study, 21 (87.5 %) showed complete resolution of clinical signs on the final visit. Fisara et al. [8] conducted an open study to assess the clinical response in 20 FAD affected dogs in Australia over a 12-week period following a single oral fluralaner treatment [8]. All clinical signs of FAD in dogs included in this study had resolved at the final assessment. In the two last studies, the assessment of dogs with FAD was based on canine atopic dermatitis extent and severity index version 3 (CADESI-03) [8, 13]. The CADESI-03 scale is validated to score canine atopic disease skin lesions and is recommended for use in clinical trials of atopic dogs, but not for FAD. In the present study, a specific scoring system (SSCFAD) was used to assess the progression of clinical signs over the course of the trial. However, this scoring system, based on Laffort-Dassot et al. [9], has not been tested for validity, reliability (i.e. inter- and intra-observer reliability and internal consistency), and responsiveness (i.e. sensitivity to change). In the present study, 18 of the 20 dogs (90 %) presented on day 84 and 15 of the 16 dogs (94 %) presented on day 168 showed complete clinical resolution.

Assessment of the severity of pruritus in dogs is critical and difficult. Various methods have been described but, to date, only one has been validated: the Pruritus Visual Analog Scale (PVAS) [11]. This scale was used in two out of three previous studies about FAD in dogs [8, 13]. In the present study PVAS was confirmed to be an easy and repeatable method for owners.

FAD was diagnosed in affected dogs on the basis of observed compatible skin lesions at typical body locations, and by exclusion of other primary pruritic skin diseases. Dogs were not skin tested with flea allergens. Some authors [8, 10, 13] used intradermal injection of flea antigens or serology as diagnostic criteria for FAD. However, positive immediate intradermal flea antigen reactivity can be observed in normal dogs, and Kunkle et al. [14] reported that 24 % of dogs can show a false-positive reaction to flea extract injections. Laffort et al. [9] demonstrated that skin testing with pure flea saliva provided the best correlation between the clinical approach to FAD diagnosis and intradermal testing, with a sensitivity of 93 %, a specificity of 90 %, and an overall accuracy of 91 % [9].

Several different insecticides (and combinations) are known to be effective against fleas and can be used to control the clinical signs of FAD. In order to deliver the best possible control of flea allergy, the antiparasitic drug should combine two major characteristics: (i) a quick flea adulticide activity; and (ii) a long duration of action with persistent efficacy. Fluralaner meets both criteria and is currently the only isoxazoline to deliver flea insecticidal efficacy >95 % for 12 weeks [4, 5]. In the present study there was no increase of mean SSCFAD or PVAS scores at D84 or D168 suggesting that a long-term control of fleas is an effective way to manage the clinical signs of FAD in dogs.

## Conclusions

Two oral fluralaner treatments effectively controlled the clinical signs of FAD in dogs and reduced the severity of the pruritus as assessed by the dog owner over a 6 months period. Orally administered fluralaner is effective for long-term control of FAD.

## Additional files

Additional file 1: Signalment of 26 FAD-affected dogs initially enrolled in the study. (DOCX 29 kb) Additional file 2: Additional data about statistical analyses. (DOCX 131 kb)
