# Supplementary material for: Open field study on the efficacy of oral fluralaner for long-term control of flea allergy dermatitis in client-owned dogs in Ile-de-France region
Source: Parasit Vectors. 2016 Mar 23;9:174. doi: 10.1186/s13071-016-1463-z (PMC4806425; doi:10.1186/s13071-016-1463-z)
Supplement: Additional file 1: — Signalment of 26 FAD-affected dogs initially enrolled in the study. (DOCX 29 kb) [file 13071_2016_1463_MOESM1_ESM.docx]

**Table (supplementary material).** Signalment of 26 FAD-affected dogs initially enrolled in the study.

| **Breed** | **Age (years)** | **Gender (status)** | **Weight** | **Housing** | **Cohabiting animals** | **Number of fleas** | **Flea feces** | **Clinical score at D0** | **Pruritus scale at D0** |
| --- | --- | --- | --- | --- | --- | --- | --- | --- | --- |
| Griffon | 4.0 | Female (entire) | 25.2kg | Suburban house | None | 0 | no | 118 | 8.6 |
| English Bulldog | 2.0 | Male (entire) | 28.4kg | Suburban house | 1 dog, 1 cat, 3 rabbits | 3 | yes | 62 | 7.5 |
| Labrador Retriever | 10.0 | Female (sterilised) | 31.0kg | Suburban house | 1 cat | 1 | yes | 47 | 5.0 |
| Crossbred Labrador | 3.5 | Female (entire) | 25.5kg | Apartment | 1 cat | 0 | yes | 99 | 10.0 |
| Shi-tzu | 1.0 | Male (entire) | 5.0kg | Apartment | None | 0 | no | 24 | 7.5 |
| Mixed breed | 3.0 | Male (entire) | 5.0kg | Suburban house | None | 1 | no | 30 | 6.7 |
| Jack Russell Terrier | 9.0 | Male (entire) | 6.3kg | Suburban house | None | 0 | no | 46 | 4.0 |
| Crossbred Brittany Spaniel | 10.0 | Male (sterilised) | 30.6kg | Suburban house | 1 cat | 1 | yes | 72 | 6.4 |
| Shih-Tzu | 2.0 | Female (entire) | 5.3kg | Apartment | 1 cat | 0 | no | 28 | 8.0 |
| Border Collie | 2.5 | Female (unknown) | 18.4kg | Suburban house | 1 dog, 3 cats | 2 | no | 24 | 8.0 |
| Brittany | 7.0 | Female (entire) | 7.5kg | Suburban house | 5 cats | 1 | no | 53 | 7.6 |
| Poodle | 14.0 | Male (entire) | 7.5kg | Apartment | None | 0 | no | 54 | 8.5 |
| Shih-Tzu | 10.0 | Male (entire) | 12.0kg | Suburban house | None | 1 | no | 84 | 8.0 |
| Jack Russell Terrier | 6.0 | Male (entire) | 7.5kg | Apartment | None | 2 | yes | 30 | 7.5 |
| Maltese | 5.5 | Male (entire) | 7.5kg | Apartment | None | 1 | no | 54 | 6.5 |
| Westie | 13.5 | Female (sterilised) | 8.5kg | Apartment and Suburban house | 1 cat | 0 | no | 30 | 8.2 |
| Jack Russell Terrier | 12.5 | Male (entire) | 7.2kg | Apartment | None | 1 | no | 20 | 9.0 |
| Labrador Retriever | 7.0 | Female (sterilised) | 31.0kg | Suburban house | 2 cats, 2 rats | 0 | no | 88 | 9.5 |
| Schiperke | 8.0 | Female (entire) | 4.5kg | Apartment | 1 cat | 0 | no | 79 | 3.9 |
| Labrador Retriever | 15.0 | Male (entire) | 40.0kg | Apartment | None | 0 | yes | 80 | 4.0 |
| Jack Russell Terrier | 6.5 | Male (entire) | 8.0kg | Apartment | None | 0 | yes | 15 | 6.5 |
| Maltese | 5.5 | Male (entire) | 6.0kg | Suburban house | None | 0 | no | 60 | 6.7 |
| Maltese | 10.5 | Female (sterilised) | 4.8kg | Suburban house | 1 cat | 1 | no | 127 | 8.2 |
| Crossbred Griffon | ? | Female (unknown) | 21.4kg | Apartment | None | 2 | no | 30 | 5.6 |
| Maltese | 12.0 | Male (entire) | 6.3kg | Apartment | None | 1 | no | 33 | 10.0 |
| Irish Setter | 5.0 | Male (sterilised) | 26.0kg | Suburban house | 2 cats | 0 | no | 17 | 8.3 |
