# Supplementary material for: Open field study on the efficacy of oral fluralaner for long-term control of flea allergy dermatitis in client-owned dogs in Ile-de-France region
Source: Parasit Vectors. 2016 Mar 23;9:174. doi: 10.1186/s13071-016-1463-z (PMC4806425; doi:10.1186/s13071-016-1463-z)
Supplement: Additional file 2: — Additional data about statistical analyses. (DOCX 131 kb) [file 13071_2016_1463_MOESM2_ESM.docx]

**Additional data about statistical analyses**

Testing results for FAD clinical scores and Pruritus Visual Analogue Scales are presented in **table A** and **table B,** respectively.

The post-treatment FAD clinical scores on days 28, 84 and 168 were significantly different from that of the pre-treatment on day 0 (P values < 0.0001 for all 3 post-treatment days). The FAD clinical scores on post-treatment days 28, 84 and 168 were not significantly different (P = 0.3651 for the difference between day 28 and day 84; P = 0.3511 for the difference between day 28 and day 168; P = 0.9363 for the difference between day 84 and day 168) from each other.

**Table A** Hypothesis Test for the FAD Clinical Scores

| **day of study** | **day of study** | **Estimate** | **Standard Error** | **Degree of Freedom** | **t Value** | **Pr > \|t\|** |
| --- | --- | --- | --- | --- | --- | --- |
| **0** | 28 | 48.48 | 5.059 | 81.0 | 9.582 | 0.0000 |
| **0** | 84 | 53.40 | 5.257 | 81.0 | 10.158 | 0.0000 |
| **0** | 168 | 53.88 | 5.616 | 81.0 | 9.593 | 0.0000 |
| **28** | 84 | 4.92 | 5.404 | 81.0 | 0.911 | 0.3651 |
| **28** | 168 | 5.40 | 5.754 | 81.0 | 0.938 | 0.3511 |
| **84** | 168 | 0.48 | 5.928 | 81.0 | 0.080 | 0.9363 |

The post-treatment pruritus visual analogue scales on post-treatment days 28, 84 and 168 were significantly different from that of the pre-treatment (P values < 0.0001 for all 3 post-treatment days). The PVAS values on post-treatment days 84 and 168 were significantly different from that on the post-treatment day 28 (P = 0.0031 for the difference between day 28 and day 84; P = 0.0002 for the difference between day 28 and day 168), but were not significantly different from each other (P = 0.2963).

**Table B** Hypothesis Test for the Analysis of Pruritus Visual Analogue Scales

| **day of study** | **day of study** | **Estimate** | **Standard Error** | **Degree of Freedom** | **t Value** | **Pr > \|t\|** |
| --- | --- | --- | --- | --- | --- | --- |
| **0** | 28 | 3.27 | 0.586 | 81.0 | 5.592 | 0.0000 |
| **0** | 84 | 5.18 | 0.608 | 81.0 | 8.515 | 0.0000 |
| **0** | 168 | 5.90 | 0.650 | 81.0 | 9.080 | 0.0000 |
| **28** | 84 | 1.91 | 0.625 | 81.0 | 3.049 | 0.0031 |
| **28** | 168 | 2.63 | 0.666 | 81.0 | 3.946 | 0.0002 |
| **84** | 168 | 0.72 | 0.686 | 81.0 | 1.051 | 0.2963 |
